# Conformational Spread in the Flagellar Motor Switch: A Model Study

**DOI:** 10.1371/journal.pcbi.1002523

**Published:** 2012-05-24

**Authors:** Qi Ma, Dan V. Nicolau, Philip K. Maini, Richard M. Berry, Fan Bai

**Affiliations:** 1 Biodynamic Optical Imaging Center and Department of Life Sciences, Peking University, Beijing, China; 2 Department of Integrative Biology, University of California at Berkeley, Berkeley, California, United States of America; 3 Centre for Mathematical Biology, Mathematical Institute, University of Oxford, Oxford, United Kingdom; 4 Clarendon Laboratory, University of Oxford, Oxford, United Kingdom; University of Illinois at Urbana-Champaign, United States of America

## Abstract

The reliable response to weak biological signals requires that they be amplified with fidelity. In *E. coli*, the flagellar motors that control swimming can switch direction in response to very small changes in the concentration of the signaling protein CheY-P, but how this works is not well understood. A recently proposed allosteric model based on cooperative conformational spread in a ring of identical protomers seems promising as it is able to qualitatively reproduce switching, locked state behavior and Hill coefficient values measured for the rotary motor. In this paper we undertook a comprehensive simulation study to analyze the behavior of this model in detail and made predictions on three experimentally observable quantities: switch time distribution, locked state interval distribution, Hill coefficient of the switch response. We parameterized the model using experimental measurements, finding excellent agreement with published data on motor behavior. Analysis of the simulated switching dynamics revealed a mechanism for chemotactic ultrasensitivity, in which cooperativity is indispensable for realizing both coherent switching and effective amplification. These results showed how cells can combine elements of analog and digital control to produce switches that are simultaneously sensitive and reliable.

## Author Summary

Bacteria swim to find nutrients or to avoid toxins. Their swimming is powered by the rotation of flagella (hair-like structures) that act as propellers. Each flagellum is driven by a rotary molecular engine (the bacterial flagellar motor) that can rotate in either a counterclockwise or clockwise direction and switches between the two directions are frequent and rapid. Although the motor has been studied in detail, we do not understand how it is able to reliably switch direction – a critical function that gives bacteria the ability to steer. In this paper we examined a mathematical model describing how a potential gearbox in the motor might work inside a ring of identical proteins. We compared the output of this model with experimental data on switching speed and other measures of motor function, finding excellent agreement. This is an exciting finding not only because the operation of the motor itself is important, but also because protein complexes play an important and ubiquitous role in cellular signal transduction and therefore, “conformational spread” may be a widespread mechanism for signal propagation in biology.

## Introduction

Bacterial chemotaxis enables the cell to move towards favorable environments. This sensing ability relies closely on collective coordination of several operation modules in the signal transduction pathway (reviewed in [1] [2]). The first component of this system is responsible for detecting environmental signals and converting them into intracellular signals. At the surface of the cell, detection of attractants and repellents is mediated by a series of chemoreceptors in the cytoplasmic membrane, the methyl-accepting chemotaxis proteins (MCPs). The second component is the intracellular chemotactic pathway, which processes extracellular signal and converts it into one that is used to determine the behavior of the bacterial flagellar motors: the concentration of the soluble cytoplasmic protein CheY. Binding of repellents induces phosphorylation of CheY, whereas binding of attractants results in CheY dephosphorylation. At the end of the chemotactic pathway lies the final component of the system – the motor block – which changes its switching bias in response to changes in CheY-P (phosphorylated CheY) concentration. On a typical *E. coli* cell surface, there are 4–5 functioning bacterial flagellar motors. When most of the motors on the membrane spin counterclockwise (CCW), flagellar filaments form a bundle and propel the cell steadily forward; if a few motors (can be as few as one) spin clockwise (CW), flagellar filaments fly apart and the cell tumbles. Therefore the cell repeats a ‘run’-‘tumble’-‘run’ pattern to perform a biased random walk for chemotaxis in a low Reynolds number world [3].

The essential feature of the motor that allows effective chemotaxis is its ability to switch direction quickly and reliably in response to small changes in environmental conditions. Previous studies have revealed that the motor switching responds ultrasensitively to changes in intracellular CheY-P concentration: a high concentration of CheY-P in the cytoplasm of the cell stimulates more CW rotation, while a low concentration of CheY-P results in more CCW rotation. In WT *E. coli*, the cytoplasmic concentration of CheY-P is around 3 mM and the flagellar motors show stochastic reversals of rotation every second or so [4]. A small change in CheY-P concentration up or down disrupts this equilibrium and produces a large shift toward either CW or CCW rotation. The sensitivity coefficient for the change in rotational bias (time spent in CCW vs. CW) as a function of CheY-P concentration (the Hill coefficient) is ∼10 at the most sensitive part of the region of operation [5].

How the flagellar motor accomplishes this switching behavior is not fully understood, partly because structural data are difficult to obtain. It is known that CheY-P molecules interact with a ring-shaped assembly of about 34 identical FliM protein subunits and this unit is believed to be responsible for determining the direction of rotation [6] [7]. For several decades, a series of models have attempted to explain the dynamic behavior of the motor switch and identify the underlying kinetic mechanisms that control the steady state behavior of motor switches [8] [9]. Tu and Grinstein [10] used a theoretical argument to suggest that in a dynamical two-state (CW and CCW) model, temporal changes in CheY-P concentration drive the switching behavior of the motor at long time scales and produces a power-law distribution for the durations of the CCW states. Bialek et al. [11] used the bacterial motor as a model system to evaluate the noise limitation of intracellular signaling, concluding that the motor switch operates close to the theoretical limit imposed by diffusive counting noise.

A key test for any model of motor switching is the ability to explain how small changes in extracellular concentration are converted into large changes in motor output. To explain this ultrasensitivity, the possibility of cooperative binding of CheY-P to the FliM subunits of the motor switch complex has been suggested [12]. However, studies focused on this binding step [13] [14] have determined a Hill coefficient of ∼1 for it, which eliminates the possibility that the amplification is driven by cooperative CheY-P binding to the motor and suggests that a separate, post-binding step within the switch complex is responsible. Duke et al. [15] described a stochastic allosteric model that qualitatively reproduces the ultrasensitive switching and locked state behavior of the motors assuming energetic coupling between neighbor units on the FliM ring inspired by the classic Ising phase transition theory. In particular, this model can reproduce the Hill coefficient of the switch, the nonlinear dependence of rotational bias on CheY-P concentration and the equilibrium between the CW and CCW locked states. The model was based on two assumptions: (a) each subunit of the ring can exist in one of two conformations: CCW and CW state and undergoes a conformational change catalyzed by the binding of CheY-P and (b) a coupling between neighboring subunits favors a coherent configuration and this leads to the propagation of conformational changes along the ring.

Although this model is able to qualitatively reproduce the equilibrium behavior of the motor switch, further work is needed to test its ability to reproduce the dynamics of the switching behavior. Here we investigated in detail the behavior of the conformational spread switching model and its ability to reproduce measurements of locked state intervals, the Hill coefficient and other measures of motor dynamics. We then performed a parameter space search to identify the parameters required to best match experimental findings and make further predictions.

## Methods

**Figure 1:**
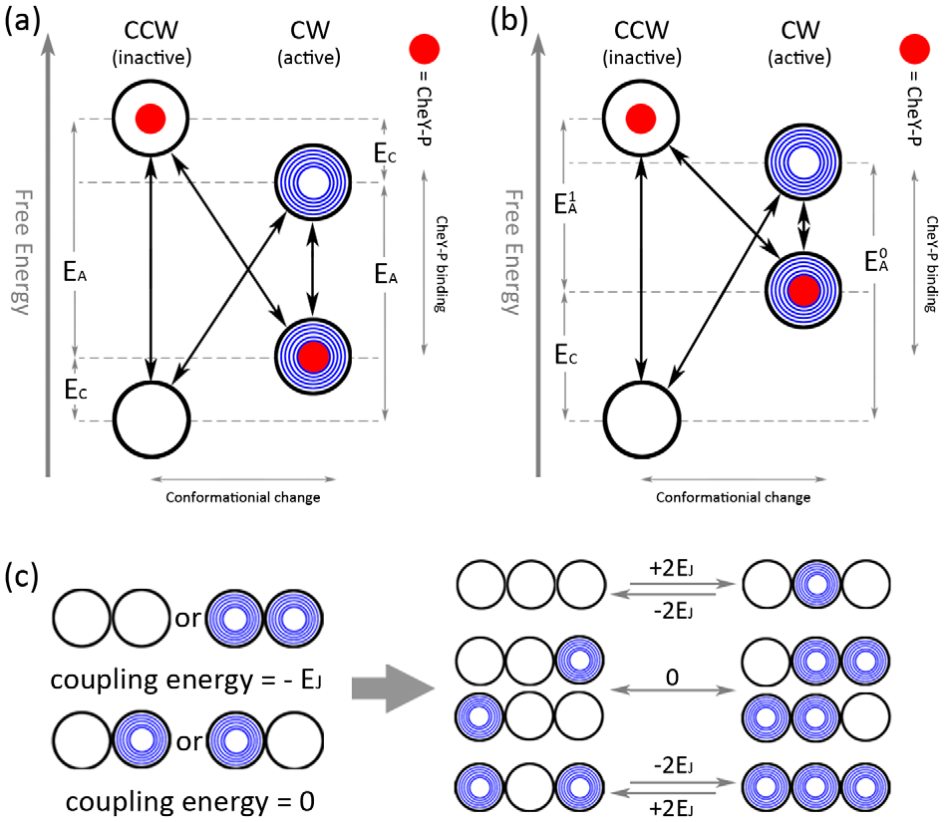
Free energy diagram for the conformational spread model a) symmectric case: the free energy of conformational change is *±E_A_*. The free energy of CheY-P binding is dependent on the CheY-P concentration as shown, with *E_C_ = −ln(c/c_0.5_)*. If *E_C_>0*, the inactive state becomes more highly populated and hence CW bias <0.5, while *E_C_<0* similarly implies CW bias >0.5. b) asymmetric case: the free energy of conformational change is *±E_A_^0^* when a protomer is unliganded and *±E_A_^1^* when it is liganded. c) coupling energy *E_J_* between neighboring protomers.

Our Monte Carlo model is based on the approach of Duke et al. [15] and our previous work [16], which we briefly describe here. In addition, we make it more general by extending the assumption of symmetry in their original model to include asymmetric cases. The centerpiece of the model is a multi-protein complex or oligomer (to simulate the FliM ring), the individual protomers of which are identical to one another and arranged in a closed ring of size 34. The interface between adjoining ring units represents domains at the boundary between proteins in a biological multi-protein complex. Each protomer can at any time be in either an active (here denoted *A* and shaded dark in Figure 1a) or inactive (here denoted *I* and left unshaded in Figure 1a) state, leading to CW and CCW rotation state, respectively. Each protomer can also be bound (here denoted *B*) or not bound (here denoted *N*) to a single CheY-P molecule. Then each protomer can make transitions between four possible states, *AB↔AN↔IN↔IB↔AB*. The model assumes that each protomer can flip reversibly between the two mechanical conformations (CW and CCW) and ligand binding/unbinding changes its chemical conformations, all of which together contribute to a free energy diagram shown in Figure 1a. To reproduce high sensitivity, the model further assumed a coupling energy between the mechanical conformations of adjacent protomers, which favors alike conformations between neighbors, but that the rate constant for CheY-P binding to a protomer is affected only by the conformation of the protomer itself.

In the original model of Duke et al. [15], it is assumed that the free energy of the active state, relative to that of the inactive state, changes from *+E_A_* to *−E_A_* when a protomer binds ligand, for simplicity (Figure 1a). In our model, to make it more general, we introduce two separate energy differences between the active and inactive states: *E_A_^0^* when a protomer is unliganded and *E_A_^1^* when it is liganded (Figure 1b).

Under the assumption of energy symmetry, the free energy change associated with CheY-P binding can be modeled as 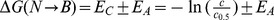, where 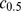 is the CheY-P concentration required for neutral bias. In the asymmetric case, we use the same definition of 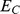, however, 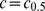 does not lead to neutral bias since 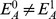. In later calculations, we use a numerical method to search for *c_0.5_*(asymmetric) as a function of 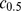. Finally, the model includes a cooperative energy term (*E_J_*, here called cooperativity) so that the free energy of a protomer is lowered by *E_J_* for each neighbor that is in the same state (Figure 1c). This interaction is crucial because it leads to the stochastic creation of semi-stable ‘domains’: regions of the ring whose constituents are either all in the active or all in the inactive state. These domains can then either (a) shrink and disappear, returning the ring to its previous coherent state or (b) grow to encompass the entire ring, a state in which it will remain until another stochastically growing domain of the opposite type will lead to another ring switch.

Given all these energy combinations, a protomer can make transitions between all possible states at rate constants describing mechanical conformational changes by:
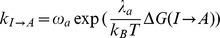


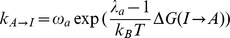


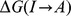
 is the sum of the free energy changes associated with changes in activity and interaction. The fundamental flipping frequency, *ω_a_*, was set as 10^4^ s^−1^, a typical rate of protein conformational change and consistent with previous modeling of the switch complex [15]. Lacking information about *λ_a_*, the parameter that specifies the degree to which changes in the free energy affect forwards as opposed to backward rate constants, an intermediate value of *λ_a_* = 0.5 was selected.

The free energy associated with CheY-P binding depends only on the conformation of the protomer bound, not on adjacent protomers. So the rate constants describing chemical conformational changes are:
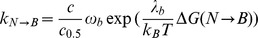


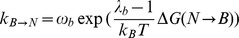
where *c* is the concentration of CheY-P, *c_0.5_* is the concentration of ligand at which protomers of the ring are 50% occupied on average under the symmetry assumption. *ω_b_* is the characteristic binding rate and *ΔG(N→B)* is the free energy associated with CheY-P binding. A value of *ω_b_* = 10 s^−1^ was selected based on the experimentally determined CheY-P binding rate [17], and consistent with previous modeling of the switch complex [15] [16], and *λ_b_* = 0 such that the binding rate is independent of protomer conformation. In the case of asymmetric *E_A_*, the CheY-P concentration corresponding to neutral bias can only be solved numerically.

We use custom written C++ code to generate a Monte-Carlo simulation of the conformational spread model [16] (including cases of both symmetric and asymmetric energy). At the beginning of each simulation, each protomer on the ring is set to active and with CheY-P bound. Later on, each protomer *n* of the ring is assigned two transition times, *An* and *Bn*, at which it will undergo a conformational change associated with (A) change between CCW and CW state and (B) CheY-P molecule binds on or off. The program progresses iteratively by locating the event in the [*A_1_, A_2……_A_34_, B_1_, B_2……_B_34_*] array with the earliest execution time, and after change its state accordingly (either mechanical state or chemical state), new transition time *An* and *Bn* of that protomer is updated by *t−t_0_ = −ln(rand)/k*, where *k* is the rate constant for the next transition, *t_0_* is the simulation time when the calculation is made and *rand* is a random number generated in the interval 0 to 1 [18]. If the transition was associated with a change in mechanical conformation of that protomer, then transition times *A*_n+1_ and *A_n−1_* for the two adjacent protomers are also recalculated (for a closed ring of protomers, we defined *A*_n+1_ for n = 34 to be A_1_ and A_n−1_ for n = 1 to be A_34_). The activity of all protomers on the ring is recorded at integer number *MΔt*, where *Δt* = 0.1 ms as the output sampling time interval and *M* goes up to 50,000,000. The algorithm continues to update protomer activities on the ring until the simulation time exceeds a specified maximum. Following Duke et al. [15], we assume that switching in the bacterial motor is controlled by the C-ring in the motor complex, which contains 34 copies of the protein FliM and therefore set *n* = 34 in our model unless otherwise stated.

In our model we have in total 4 free parameters: *E_A_^0^*, *E_A_^1^*, *E_J_*, *c*. The CheY-P concentration *c* is expressed in the unit of *c_0.5_* and when we change it we see the ring operate at different bias and therefore the response curve can be plotted. In the following sections, when we make predictions about ring switching time, switching interval etc., we searched the parameter space *E_A_^0^*, *E_A_^1^* and *E_J_* across the ranges 0.5 *k_B_T*≤*E_A_^0^*≤1.5 *k_B_T* , 0.5 *k_B_T*≤*E_A_^1^*≤1.5 *k_B_T* , and 3.5 *k_B_T*≤*E_J_*≤4.5 *k_B_T* (shown in Table 1), but for each parameter set, we only present results at neutral bias for simplicity.

**Table 1:** Model predictions about motor switching time, locked state time, and Hill coefficient with parameters *E_A_^0^*, *E_A_^1^* and *E_J_* ranging across 0.5 *k_B_T*≤*E_A_^0^*≤1.5 *k_B_T* , 0.5 *k_B_T*≤*E_A_^1^*≤1.5 *k_B_T* , and 3.5 *k_B_T*≤*E_J_*≤4.5 *k_B_T*.

E_A_^0^	E_A_^1^	E_J_	Mean Locked State time (s)	Mean Switch Time (ms)	Locked state / Switch time ratio	Hill coefficient
0.5	0.5	3.5	0.14	12.03	11.59	5.85
0.5	0.75	3.5	0.20	15.46	13.10	6.74
0.5	1	3.5	0.29	20.10	14.41	7.81
0.5	1.25	3.5	0.39	26.24	14.91	7.93
0.5	1.5	3.5	0.52	33.50	15.44	8.51
0.75	0.5	3.5	0.20	15.43	12.70	6.93
0.75	0.75	3.5	0.33	22.91	14.18	8.82
0.75	1	3.5	0.50	34.59	14.42	9.65
0.75	1.25	3.5	0.74	49.96	14.87	10.44
0.75	1.5	3.5	1.05	70.95	14.85	11.02
1	0.5	3.5	0.26	19.92	13.26	7.50
1	0.75	3.5	0.49	33.54	14.67	9.42
1	1	3.5	0.79	53.67	14.75	11.00
1	1.25	3.5	1.24	84.93	14.55	12.34
1	1.5	3.5	1.68	126.10	13.31	12.60
1.25	0.5	3.5	0.34	24.99	13.61	8.03
1.25	0.75	3.5	0.67	47.57	14.09	10.37
1.25	1	3.5	1.18	82.97	14.28	12.17
1.25	1.25	3.5	1.79	136.45	13.11	12.91
1.25	1.5	3.5	2.40	196.82	12.22	13.68
1.5	0.5	3.5	0.42	30.84	13.52	8.46
1.5	0.75	3.5	0.86	62.97	13.67	10.92
1.5	1	3.5	1.56	114.94	13.55	12.71
1.5	1.25	3.5	2.43	188.54	12.89	13.98
1.5	1.5	3.5	3.48	269.46	12.93	14.49
0.5	0.5	3.75	0.22	12.56	17.13	6.57
0.5	0.75	3.75	0.31	16.17	19.37	7.75
0.5	1	3.75	0.44	20.82	21.13	8.51
0.5	1.25	3.75	0.59	27.03	21.93	8.92
0.5	1.5	3.75	0.78	35.54	21.95	8.93
0.75	0.5	3.75	0.30	16.28	18.64	7.80
0.75	0.75	3.75	0.50	24.08	20.87	9.76
0.75	1	3.75	0.78	36.42	21.30	10.60
0.75	1.25	3.75	1.11	51.74	21.51	11.59
0.75	1.5	3.75	1.54	71.87	21.44	11.70
1	0.5	3.75	0.41	20.38	19.91	8.54
1	0.75	3.75	0.73	34.65	21.10	10.82
1	1	3.75	1.22	57.97	21.00	12.55
1	1.25	3.75	1.84	88.26	20.89	13.91
1	1.5	3.75	2.61	130.34	20.05	14.34
1.25	0.5	3.75	0.52	26.50	19.63	9.19
1.25	0.75	3.75	1.02	48.57	21.04	12.00
1.25	1	3.75	1.77	85.60	20.68	13.62
1.25	1.25	3.75	2.63	136.31	19.32	14.65
1.25	1.5	3.75	3.88	199.85	19.40	16.06
1.5	0.5	3.75	0.64	32.20	19.83	9.38
1.5	0.75	3.75	1.31	66.24	19.73	12.05
1.5	1	3.75	2.27	114.87	19.80	14.28
1.5	1.25	3.75	3.70	193.35	19.16	15.69
1.5	1.5	3.75	5.26	279.81	18.78	16.05
0.5	0.5	4	0.33	12.73	26.26	7.16
0.5	0.75	4	0.49	16.72	29.56	8.48
0.5	1	4	0.70	21.56	32.50	9.28
0.5	1.25	4	0.95	28.07	33.76	9.65
0.5	1.5	4	1.21	35.53	34.12	9.81
0.75	0.5	4	0.46	16.75	27.71	8.60
0.75	0.75	4	0.78	25.13	30.93	10.38
0.75	1	4	1.23	37.11	33.09	11.47
0.75	1.25	4	1.76	53.77	32.79	12.87
0.75	1.5	4	2.38	77.16	30.82	13.11
1	0.5	4	0.63	21.44	29.40	9.36
1	0.75	4	1.16	35.94	32.38	11.94
1	1	4	1.91	59.82	31.88	13.35
1	1.25	4	2.93	92.14	31.76	14.84
1	1.5	4	4.14	132.32	31.28	15.67
1.25	0.5	4	0.79	27.10	29.11	10.08
1.25	0.75	4	1.64	51.49	31.91	12.87
1.25	1	4	2.64	88.80	29.77	14.70
1.25	1.25	4	4.24	140.88	30.10	16.14
1.25	1.5	4	6.15	205.06	29.98	16.44
1.5	0.5	4	1.00	33.51	29.80	10.22
1.5	0.75	4	2.18	67.85	32.11	13.43
1.5	1	4	3.64	119.57	30.41	15.36
1.5	1.25	4	5.60	194.46	28.81	16.82
1.5	1.5	4	8.26	295.79	27.92	18.89
0.5	0.5	4.25	0.55	12.97	42.29	7.47
0.5	0.75	4.25	0.79	17.08	46.10	8.92
0.5	1	4.25	1.09	21.74	50.10	9.65
0.5	1.25	4.25	1.51	28.51	53.01	10.19
0.5	1.5	4.25	1.93	37.39	51.68	10.49
0.75	0.5	4.25	0.74	16.79	44.27	8.91
0.75	0.75	4.25	1.25	25.80	48.34	11.03
0.75	1	4.25	1.92	39.11	49.00	12.32
0.75	1.25	4.25	2.78	55.11	50.48	12.99
0.75	1.5	4.25	3.76	76.61	49.08	14.14
1	0.5	4.25	1.02	22.03	46.22	9.62
1	0.75	4.25	1.81	37.06	48.73	12.23
1	1	4.25	3.09	63.37	48.83	14.45
1	1.25	4.25	4.89	96.96	50.47	15.50
1	1.5	4.25	6.69	140.42	47.62	16.00
1.25	0.5	4.25	1.30	27.52	47.20	10.55
1.25	0.75	4.25	2.54	52.73	48.11	13.31
1.25	1	4.25	4.35	95.35	45.64	15.27
1.25	1.25	4.25	6.87	152.84	44.98	17.62
1.25	1.5	4.25	9.71	216.86	44.79	18.81
1.5	0.5	4.25	1.58	34.53	45.73	10.94
1.5	0.75	4.25	3.32	68.00	48.78	14.00
1.5	1	4.25	5.98	125.94	47.48	16.22
1.5	1.25	4.25	9.05	199.17	45.46	18.36
1.5	1.5	4.25	13.10	293.22	44.68	18.96
0.5	0.5	4.5	0.87	13.55	64.10	7.75
0.5	0.75	4.5	1.26	17.34	72.56	9.24
0.5	1	4.5	1.78	23.17	76.80	10.06
0.5	1.25	4.5	2.42	29.00	83.59	10.57
0.5	1.5	4.5	3.19	38.71	82.51	11.52
0.75	0.5	4.5	1.22	17.45	69.70	9.38
0.75	0.75	4.5	2.12	26.46	80.13	11.45
0.75	1	4.5	3.20	38.81	82.45	12.63
0.75	1.25	4.5	4.73	56.85	83.24	13.63
0.75	1.5	4.5	6.56	79.96	82.08	14.17
1	0.5	4.5	1.67	22.14	75.36	10.39
1	0.75	4.5	3.04	37.58	80.94	13.03
1	1	4.5	4.95	62.47	79.28	14.86
1	1.25	4.5	7.46	99.65	74.84	16.36
1	1.5	4.5	10.14	138.80	73.05	17.31
1.25	0.5	4.5	2.12	27.77	76.34	10.88
1.25	0.75	4.5	4.19	54.35	77.05	14.04
1.25	1	4.5	7.47	101.29	73.75	16.34
1.25	1.25	4.5	11.61	149.58	77.62	17.20
1.25	1.5	4.5	14.87	215.64	68.97	19.58
1.5	0.5	4.5	2.61	34.56	75.58	11.24
1.5	0.75	4.5	5.48	70.02	78.22	14.52
1.5	1	4.5	9.68	131.07	73.82	17.09
1.5	1.25	4.5	15.37	208.46	73.72	19.58
1.5	1.5	4.5	22.49	322.65	69.71	20.63

## Results

### Random patterns and domains on the ring

**Figure 2:**
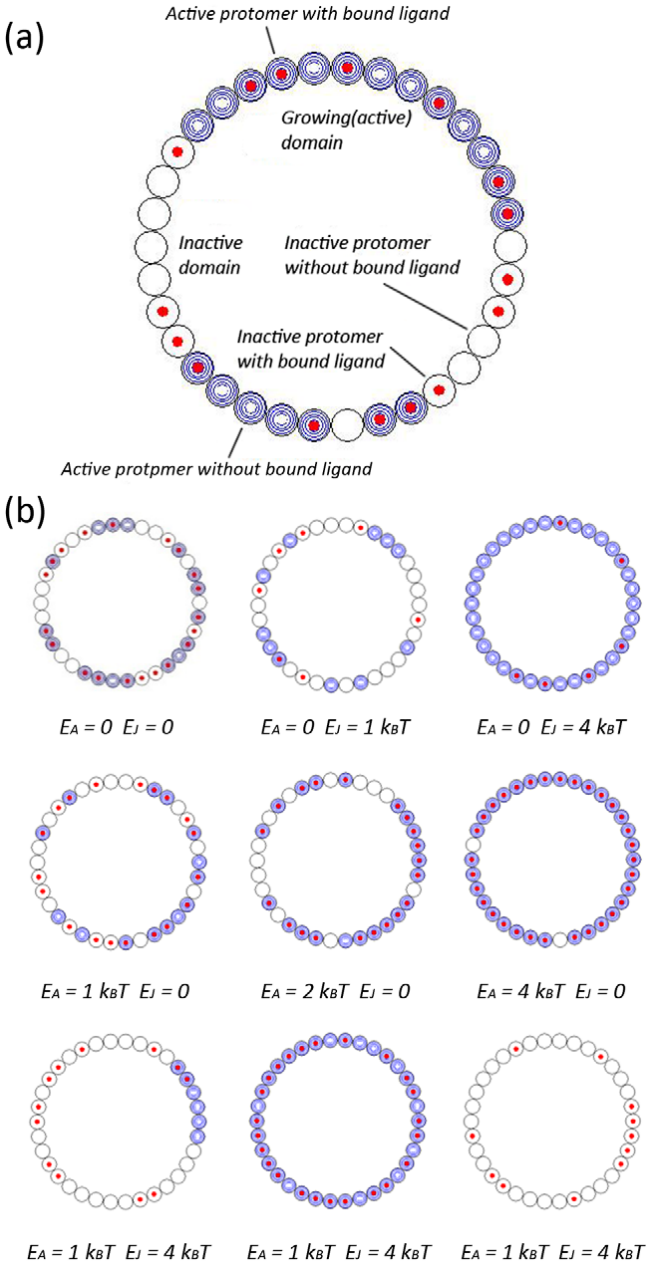
Snapshots of ring activities with different model parameters. a) Ring activity with symbols showing mechanical and chemical conformations of each protomer and multiple quasi-stable domains. b) Typical ring state images for *E_A_* = 0 (top), *E_J_* = 0 (middle) and *E_A_* = 1*k_B_T*, *E_J_* = 4*k_B_T* (bottom).

A typical screenshot of the ring with multiple domains, labeled with a symbol legend, is shown in Figure 2a. We simulated the qualitative behavior of the ring for a few carefully chosen special cases under the symmetry assumption. Typical screenshots of the ring representing different regimes in the parameter space are shown in Figure 2b.

If the activation energy is zero (*E_A_* = 0, Figure 2b, top row), growing domains can only form at random through cooperativity between neighbors, but are unstable and unable to grow sufficiently quickly to encompass the ring because any one of their constituent protomers has a high probability of flipping. When the cooperativity energy becomes high, a coherent ring conformation starts to emerge as the coupling between neighboring protomers is sufficiently strong to lock the whole ring in one conformation. However, as the activation energy is zero, the switch complex loses its ability to respond to ligand concentration changes and switching between coherent inactive and coherent active states can be very slow.

In the absence of cooperativity (*E_J_* = 0, Figure 2b, middle row), the ring displays random salt-and-pepper patterns reflecting the underlying stochasticity of the ligand binding and unbinding process. When the activation energy becomes high, absolute coupling between chemical conformation and mechanical conformation starts to emerge, and the protomers exist in inactive form only when there is no ligand bound and change to active form once ligand binds. In this case, a coherent active conformation of the ring only exists when there are 34 ligands bound to the ring and for a coherent inactive conformation of the ring we find 0 ligand bound.

When cooperativity and activation energy are both present at appropriate magnitudes (*E_A_* = 1 *k_B_T*, *E_J_* = 4 *k_B_T* as discussed in reference [15], Figure 2b, bottom row), the ring spends most of its time locked in either a coherent inactive or active conformation, with transitions between the two (switches) accomplished rapidly by means of a spreading domain. In order to achieve both ring stability and coherent switching, cooperativity is needed to ensure the presence and growth of domains, and activation energy is needed to stabilize these domains by ligand binding. In this energy regime of the model parameter space, the conformational spread model best simulate the performance of the flagellar motor switching responding to external signals.

*Ring activity, locked states and switching.*

**Figure 3:**
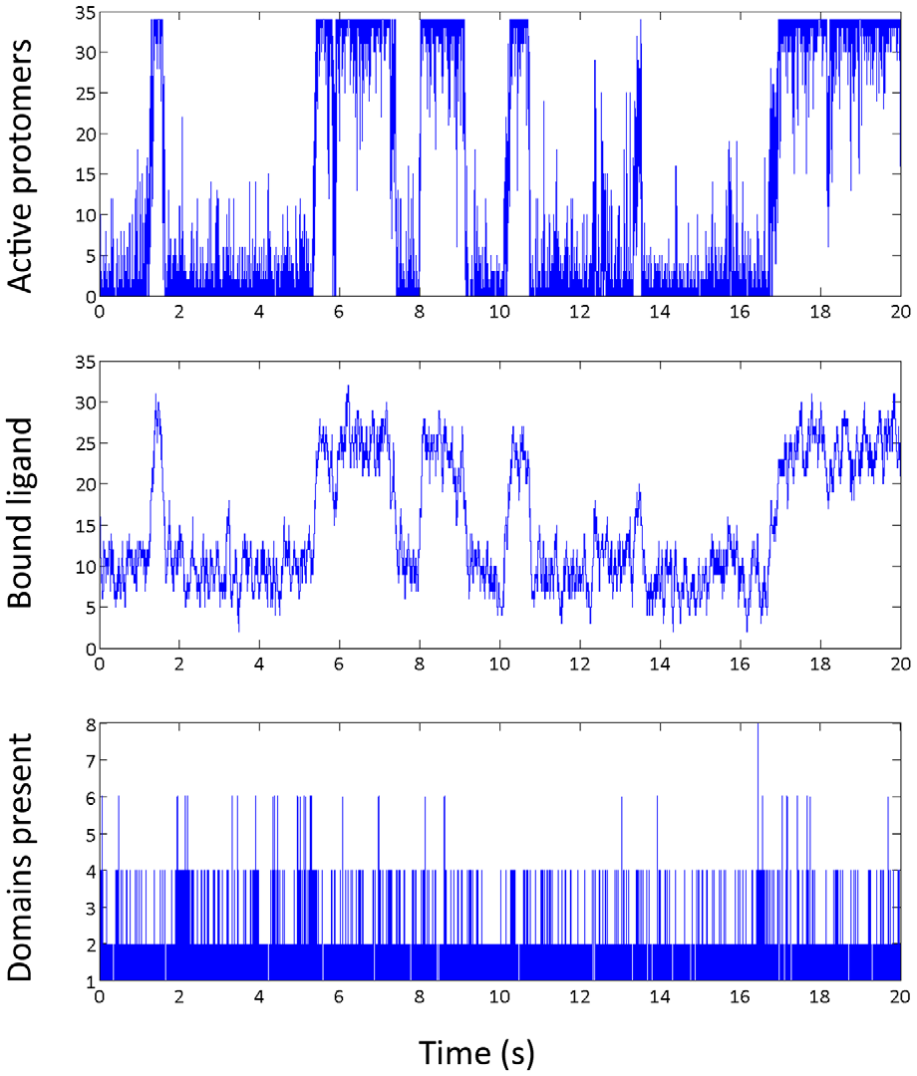
Activity of the ring at high cooperativity and low activation energy. Top: number of active protomers, showing locked state behaviour (0 and 34 protomers, respectively) and rapid switching. Middle: number of protomers with bound ligand; note that this corresponds closely to the number of active protomers (top panel). Bottom: the number of individual domains (of the opposite state to the current locked state); there are almost never more than two domains, even during switching events. Switching events are often, but not always, associated with two domains fusing.

We simulated the behavior of the 34-protomer ring with the method introduced earlier and *E_A_^0^* = *E_A_^1^* = 1 *k_B_T*, *E_J_* = 4 *k_B_T*. A typical result is shown in Figure 3. The top panel is a time series of the number of active ring protomers (34 active protomers correspond to the CW state and 0 to CCW in our convention). The middle panel shows the number of protomers with bound ligand. This graph matches that of the locked states (i.e. the two fit on top of each other if superimposed) along both axes: ligand binding makes the active state more favorable and the active state binds ligand more strongly and the two effects cooperate to produce locked state and switching behavior.

The bottom panel shows the number of independent domains (see Figure 2a for an illustration of typical domain formation present on the ring at any time). Domains appear within a locked CCW or CW state because of stochastic flipping events in protomers and their growth is driven by ligand binding and unbinding and protomer-protomer cooperativity. The domains are transient features of the ring's behavior. They can either (a) disappear or (b) grow (alone or by fusing with nearby domains) to encompass the whole ring, with these latter events corresponding to switches and occurring very rarely: <1% of domains lead to a switch. We find that at the parameter values identified by Duke et al. [15], the number of independent domains almost never exceed 6 (and that such a ring state only exists ∼0.0279% of the time). The vast majority of the time, the ring either contains one domain (coherent state, 83.77% of the time) or contains two domains (15.34% of the time). Four domains are present 0.8685% of the time.

### The model reproduces the sensitivity of the rotary motor

In addition to being able to reproduce the locked coherent state on the ring and fast switching behavior, a separate key test of the conformational spread model is its ability to reproduce the relationship between changes in CheY-P concentration and motor bias. The Hill coefficient (the maximum sensitivity of the switch) is defined in this case using the relation:
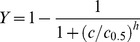
where *Y* is the CW bias, *h* is the Hill coefficient, *c* is the concentration of the CheY-P and *c_0.5_* is the concentration required for neutral bias (in the case of asymmetric model, replace *c_0.5_* to *c_0.5_*(asymmetric)). Here we estimated the Hill coefficient by fitting a linear equation to a plot of log[*Y/(1−Y)*] against log(*c/c*_0.5_), with the slope of this line corresponding to *h*. For each parameter set, its characteristic Hill curve can be generated by long time simulation with varying *c*, and plot CW bias of the simulated trace as a function of *c*.

The sensitivity of ring activity to changes in ligand concentration depends more strongly on the activation energy and considerably less on the cooperativity. A lower sensitivity can be brought about by a lower activation energy or by a lower cooperativity, with the activation energy having the dominating influence. However, with a 34 protomer ring, the cooperativity can be no less than the critical cooperativity required for coherent switching to occur, i.e. *E_J_*>3.5 *k_B_T* [15]. In table 1, we present the Hill coefficient calculated for parameters *E_A_^0^*, *E_A_^1^* and *E_J_* across the ranges 0.5 *k_B_T*≤*E_A_^0^*≤1.5 *k_B_T* , 0.5 *k_B_T*≤*E_A_^1^*≤1.5 *k_B_T* , and 3.5 *k_B_T*≤*E_J_*≤4.5 *k_B_T*. We see that the experimentally determined Hill coefficient ∼10 can be reproduced by a large parameter sets.

### Distributions of locked state intervals

The behavioral features of the ring can be further characterized by the distributions of (a) the times spent in the two locked states and (b) the times required for both CCW→CW and CW→CCW switches. Here we used simulated ring state data to obtain the theoretical length of the locked state intervals predicted by the model. Because we have direct access to the fundamental protomer states, filtering and threshold algorithms are not needed to identify the intervals (and switches, respectively). Because transitions between the two locked states are not instantaneous, we needed an unambiguous way to define CCW and CW intervals, respectively. We defined such an interval as the time (in simulation steps) between when the ring enters a fully locked state (0 or 34 active protomers, respectively) and when it next enters the other fully locked state (i.e. 34 or 0 active protomers, respectively).

**Figure 4:**
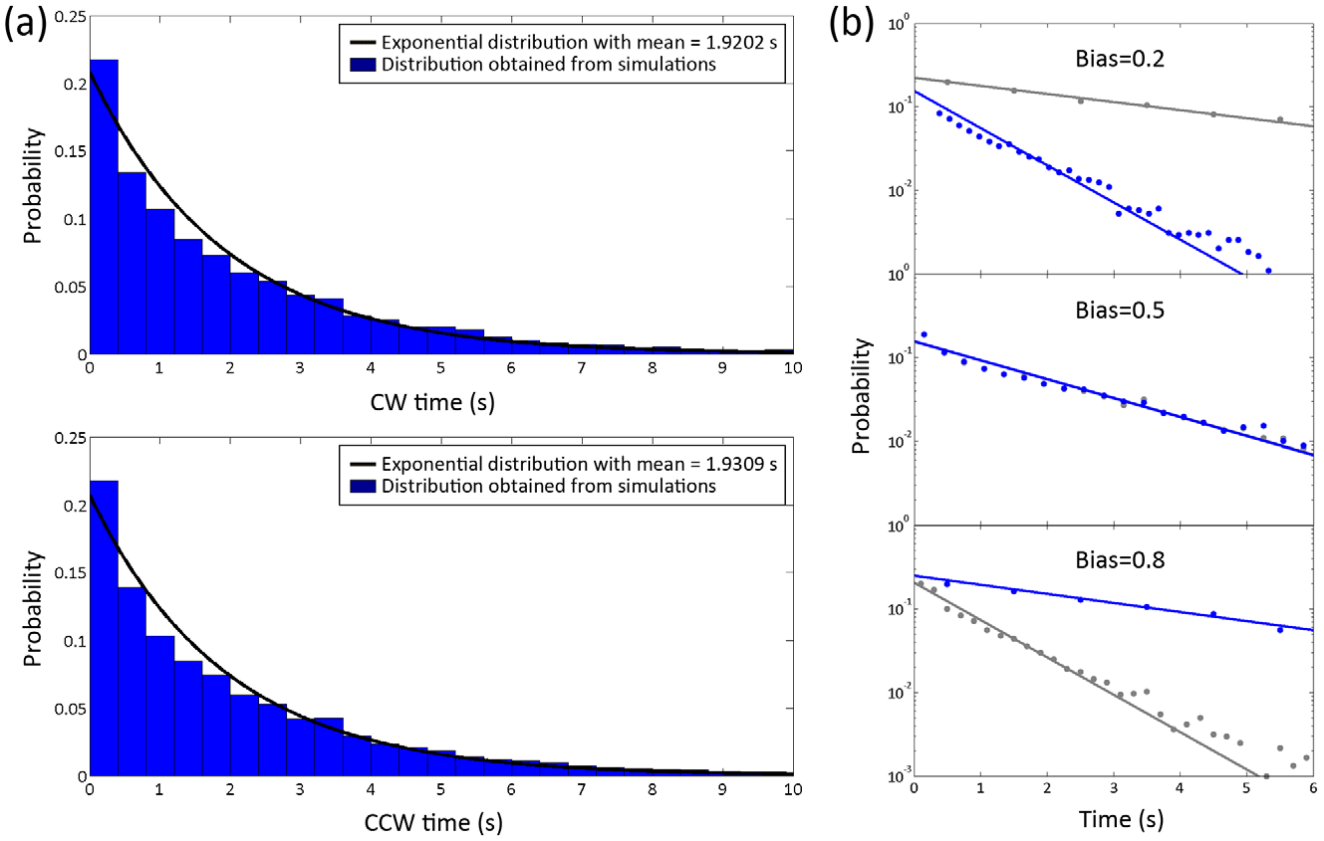
Distributions of times spent in the locked CCW and CW states. (a) Distributions of the locked state interval at a bias of 0.5 (*E_A_^0^* = *E_A_^1^* = 1 *k_B_T*, *E_J_* = 4*k_B_T*). Exponential fitting to the histogram is shown overlaid by the black line. (b) Distributions of the locked state intervals at different bias values (0.2, 0.5, 0.8). Lines are exponential fit on a log-linear axes, while the fitting of CW, CCW times are shown in blue and grey, respectively.

Distributions of locked state intervals obtained from simulation traces (*E_A_^0^* = *E_A_^1^* = 1 *k_B_T* and *E_J_* = 4 *k_B_T* at neutral bias) equivalent to 30000 seconds of real time are shown in Figure 4 (a). To make a comparison, the log-linear plot of the distributions at low (0.2) and high (0.8) CW biases are also shown in Figure 4(b). Least-squares fitting of exponential curves to the simulation data are shown overlaid. We see that the locked state distribution follows an exponential distribution. In table 1, we presented the mean locked state interval values calculated for parameter *E_A_^0^*, *E_A_^1^* and *E_J_* across the ranges 0.5 *k_B_T*≤*E_A_^0^*≤1.5 *k_B_T* , 0.5 *k_B_T*≤*E_A_^1^*≤1.5 *k_B_T* , and 3.5 *k_B_T*≤*E_J_*≤4.5 *k_B_T*. Within the parameter range of our simulation, the minimum value of mean locked state time is 0.13 s and the maximum value is 22.17 s (shown in Table 1). The mean locked state time increases when the energy of activation or cooperativity is increased, with the activation energy *E_A_* having the dominant influence.

### Distributions of switch times

The essential feature of interest of the model proposed by Duke et al. [15] is that the ring can simultaneously achieve very rapid switches and very stable locked states. This qualitatively matches what is observed in the rotary motors of flagellar bacteria such as *E. coli*, which can rotate at hundreds of RPM stably for a long period but switch direction quickly (on the order of ms) and stochastically. Distinct from the classic MWC model, which requires coherent switches to happen instantaneously, in our model switches occur by a mechanism of conformational spread. We defined a switch time as the time (in simulation steps) between when the ring leaves a fully locked state (0 or 34 active protomers, respectively) and when it next enters the other fully locked state (i.e. 34 or 0 active protomers, respectively).

**Figure 5:**
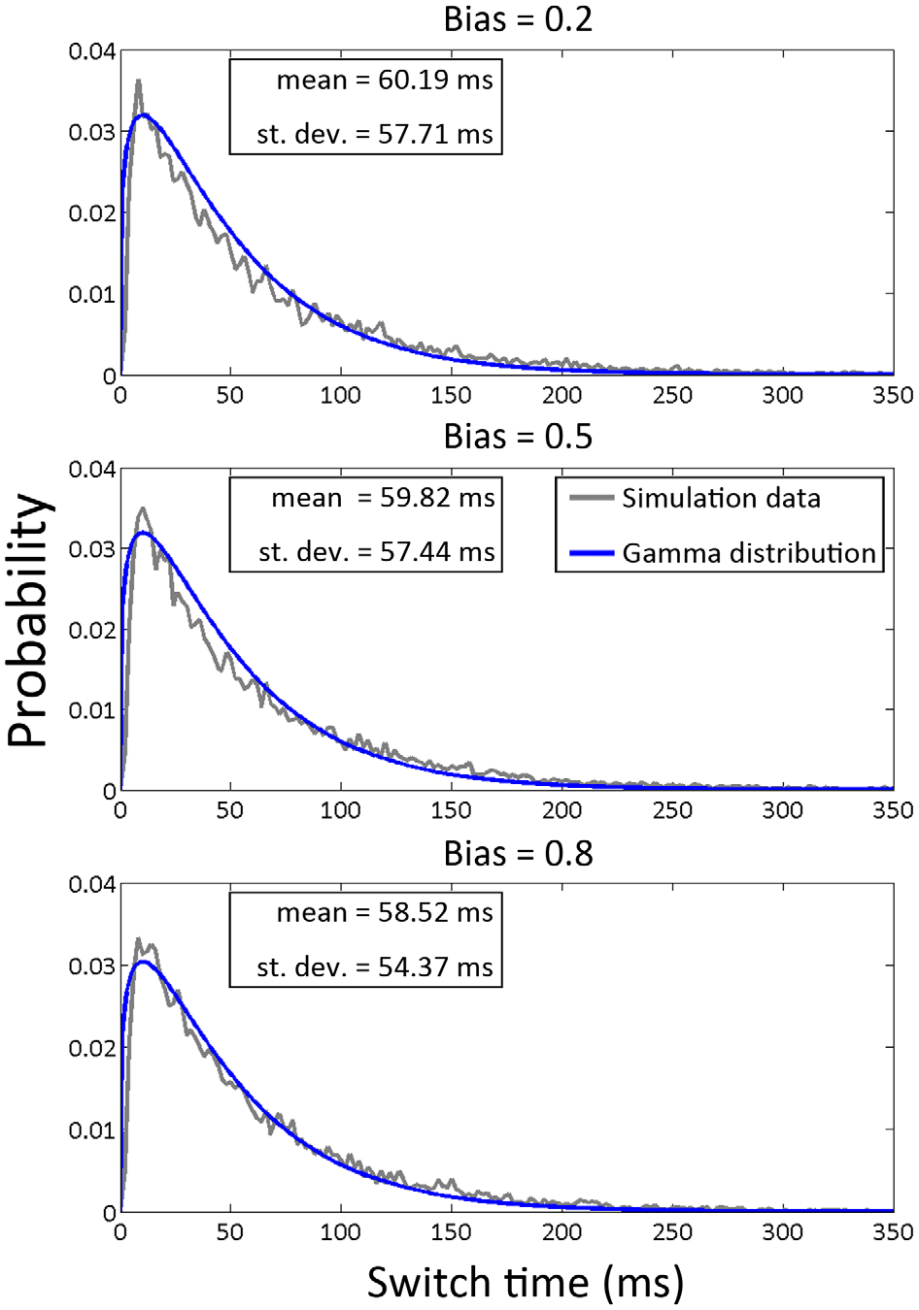
Histograms of switch times for low (0.2), middle (0.5) and high (0.8) CW bias. The histograms follow gamma distributions with means of ∼58–61 ms and standard deviations of ∼54–58 ms.

We simulated the behavior of the ring at the optimal activation energy and cooperativity values identified earlier (*E_A_^0^* = *E_A_^1^* = 1 *k_B_T*, *E_J_* = 4 *k_B_T*) for different values of bias. The empirical distributions thus determined are shown in Figure 5.

In contrast to the distributions of locked state intervals, the switch times follow a peaked gamma distribution. At the parameter value chosen, the mean lies between 58–61 ms for low (0.2), middle (0.5) and high (0.8) biases and these are statistically independent of bias and of direction of switch. In table 1, we presented the mean switch time values calculated for parameter *E_A_^0^*, *E_A_^1^* and *E_J_* across the ranges 0.5 *k_B_T*≤*E_A_^0^*≤1.5 *k_B_T* , 0.5 *k_B_T*≤*E_A_^1^*≤1.5 *k_B_T* , and 3.5 *k_B_T*≤*E_J_*≤4.5 *k_B_T*. The result of our simulation shows that the mean switch time values changes across the ranges from 12.03 ms to 322.65 ms. The mean switch time increases when activation or cooperativity energy is increased, with the activation energy having the dominant influence.

### Power spectra

To confirm that typically one domain of opposite conformation (rather than several) grows to encompass the entire ring, we also computed power spectra for the ring activity traces in order to characterize the spectral properties of the ring switch complex. If switching events are associated with a single nucleation event (a Possion step), we expect the power spectra of the trace to be monotonically decreasing with a ‘knee’, i.e. display a Lorenzian profile. In contrast, if switching events are associated with multiple hidden steps, as for example in a closed biochemical system with hidden reactions, then we expect a non-Lorentzian profile with a peak (a local maximum) at a characteristic frequency related to the number of steps involved [19].

**Figure 6:**
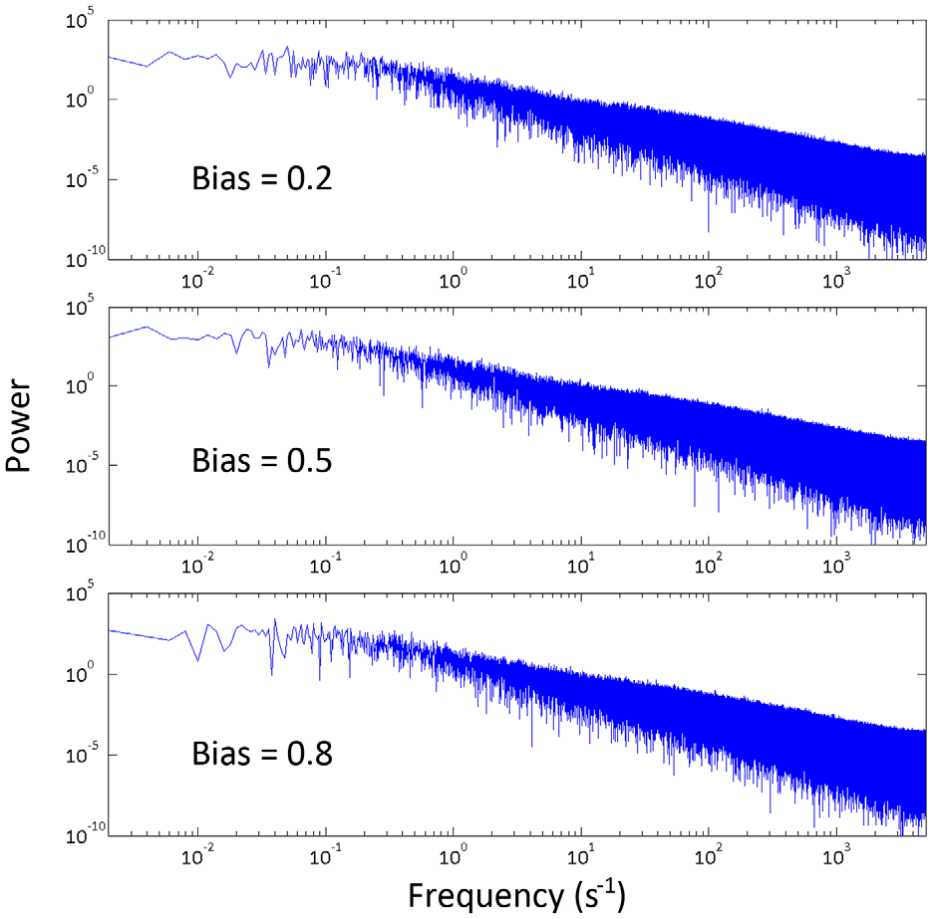
Power spectra showing a Lorentzian profile at different values of bias, consistent with one single nucleation growing to encompass the ring during a switch.

Our simulation results (Figure 6) show the spectra thus obtained are Lorentzian without a local maximum at long times. This behavior is observed at different values of bias. These results offer an internal confirmation of the model results shown in Figure 4, which indicate that the distributions of times spent in the locked states are exponential. Such a system would be expected to display power spectra with Lorentzian profiles. However, because the power spectra and locked state time distributions are computed independently and by different methods, the result that they predict the same behavior is an important internal test of the model. In particular, the power spectra results confirm that the locked state time distributions are not an artifact of our algorithm for detecting the start and end of a locked state.

### Parameterize the model using experimental measurements

In our recent experimental paper [16], we used a high-resolution optical system to measure the switching time and locked state interval of bacterial flagellar motors. The experimental observations confirmed that the switching time distribution follows a broad gamma distribution with mean switch time 18.72 ms and the locked state interval follows an exponential distribution with mean interval value 0.75 s at neutral bias. Hence we use mean switch time (∼18 ms), mean locked state time (∼0.75 s) and Hill coefficient (∼10) to parameterize our model.

**Figure 7:**
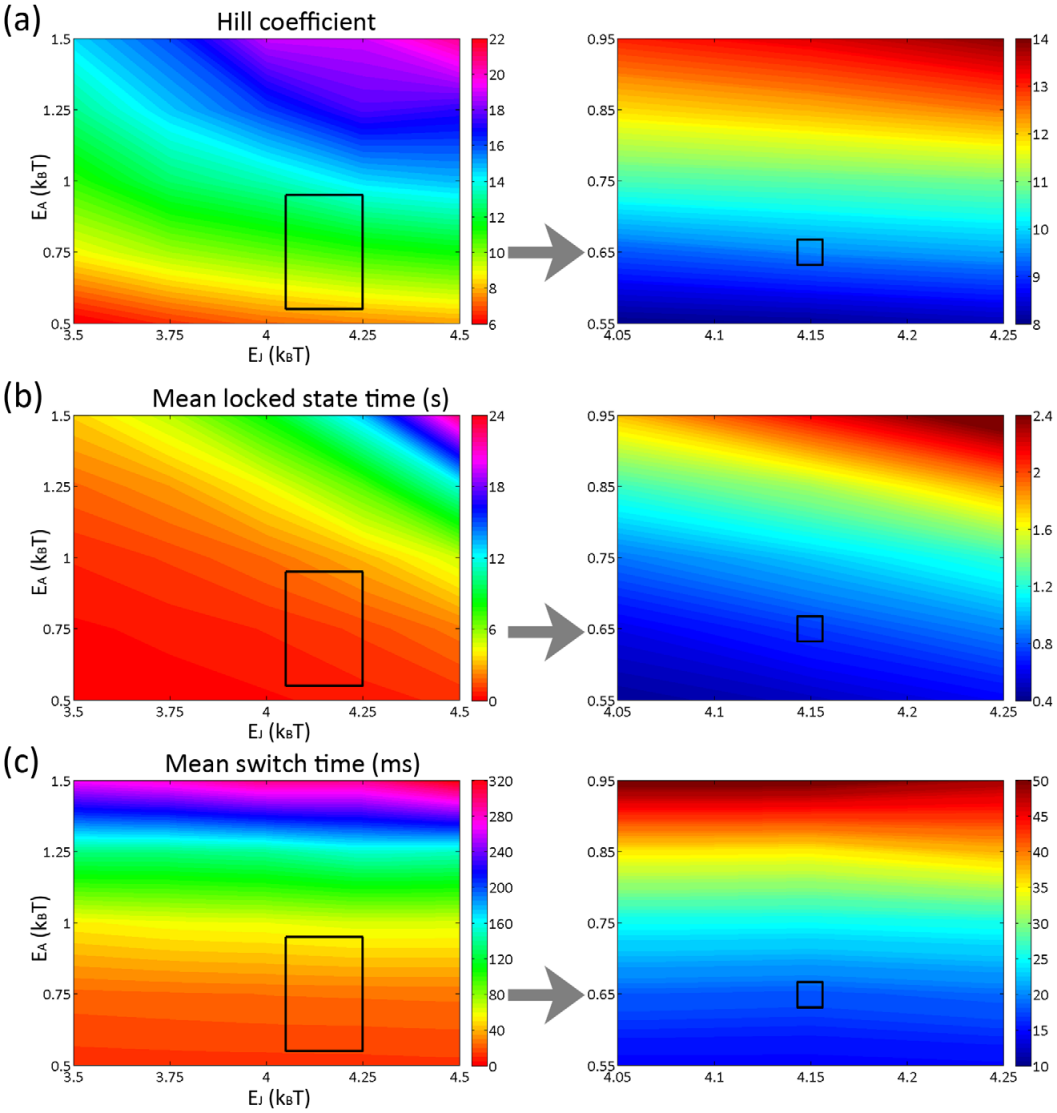
Two dimensional contour plot showing A) Hill coefficient B) Mean locked state time C) Mean switch time as a function of activation energy *E_A_* and coupling energy *E_J_* of the conformational spread model. Left column: simulation result with 0.5 *k_B_T*≤*E_A_*≤1.5 *k_B_T* and 3.5 *k_B_T*≤*E_J_*≤4.5 *k_B_T*. The rectangular region has been selected for a fine parameter search. Right column: simulation result with 0.55 *k_B_T*≤*E_A_*≤0.95 *k_B_T* and 4.05 *k_B_T*≤*E_J_*≤4.25 *k_B_T*. The best-fit parameter set has been labeled by a square.

Table 1 shows a coarse parameter search of our model with predictions of the switching time, locked state interval, and Hill coefficient. We identify the 0.55 *k_B_T*≤*E_A_^0^*≤0.95 *k_B_T* , 0.55 *k_B_T*≤*E_A_^1^*≤0.95 *k_B_T* , and 4.05 *k_B_T*≤*E_J_*≤4.25 *k_B_T* region for a fine parameter search (Table 2). We see with only a few parameter sets, the conformational spread model is able to reproduce the three experimentally determined quantities. With the results shown in Table 2, we find 3 groups of values that fit the experimental values determined by Bai et al. [16]. They are *E_A_^0^ = 0.55k_B_T E_A_^1^ = 0.75k_B_T E_J_ = 4.15 k_B_T, E_A_^0^ = 0.75k_B_T E_A_^1^ = 0.55k_B_T E_J_ = 4.15 k_B_T, E_A_^0^ = E_A_^1^ = 0.65k_B_T E_J_ = 4.15 k_B_T*. We therefore expect a conformational spread model with activation energy ∼*0.65 k_B_T* and coupling energy ∼*4.15 k_B_T* can well reproduce experimental observations. Please see Figure 7 for a visual summary of our computational results. For simplicity, we only showed those values with *E_A_^0^ = E_A_^1^ = E_A_* and the best-fit parameter set has been labeled by a square.

**Table 2:** Model predictions about motor switching time, locked state time, and Hill coefficient with parameters *E_A_^0^*, *E_A_^1^* and *E_J_* ranging across 0.55 *k_B_T*≤*E_A_^0^*≤0.95 *k_B_T* , 0.55 *k_B_T*≤*E_A_^1^*≤0.95 *k_B_T* , and 4.05 *k_B_T*≤*E_J_*≤4.25 *k_B_T*.

E_A_^0^	E_A_^1^	E_J_	Mean Locked State time (s)	Mean Switch Time (ms)	Locked state / Switch time ratio	Hill coefficient
0.55	0.55	4.05	0.44	14.44	30.39	8.00
0.55	0.65	4.05	0.50	16.01	31.09	8.52
0.55	0.75	4.05	0.59	18.13	32.79	9.03
0.55	0.85	4.05	0.68	20.21	33.59	9.31
0.55	0.95	4.05	0.80	22.55	35.41	10.01
0.65	0.55	4.05	0.50	16.08	31.07	8.57
0.65	0.65	4.05	0.59	18.51	32.07	9.22
0.65	0.75	4.05	0.75	21.22	35.14	9.89
0.65	0.85	4.05	0.86	24.51	35.24	10.26
0.65	0.95	4.05	1.01	28.34	35.62	10.85
0.75	0.55	4.05	0.59	18.14	32.29	9.10
0.75	0.65	4.05	0.72	21.48	33.46	10.02
0.75	0.75	4.05	0.86	25.19	34.32	10.66
0.75	0.85	4.05	1.03	28.95	35.58	11.29
0.75	0.95	4.05	1.23	35.87	34.33	11.67
0.85	0.55	4.05	0.65	20.10	32.29	9.50
0.85	0.65	4.05	0.83	24.51	33.95	10.53
0.85	0.75	4.05	0.97	30.01	32.41	11.27
0.85	0.85	4.05	1.26	35.64	35.28	11.91
0.85	0.95	4.05	1.48	41.85	35.36	12.46
0.95	0.55	4.05	0.73	23.04	31.55	9.95
0.95	0.65	4.05	0.94	27.62	34.18	10.78
0.95	0.75	4.05	1.17	35.06	33.27	11.80
0.95	0.85	4.05	1.45	41.29	35.03	12.66
0.95	0.95	4.05	1.76	50.99	34.58	13.06
0.55	0.55	4.15	0.52	14.40	35.75	8.09
0.55	0.65	4.15	0.62	16.04	38.33	8.72
0.55	0.75	4.15	0.74	18.32	40.16	9.27
0.55	0.85	4.15	0.82	20.13	40.79	9.71
0.55	0.95	4.15	0.96	23.15	41.65	10.13
0.65	0.55	4.15	0.61	16.40	37.13	8.74
0.65	0.65	4.15	0.74	18.17	40.98	9.52
0.65	0.75	4.15	0.87	21.69	40.10	10.10
0.65	0.85	4.15	1.04	24.62	42.19	10.73
0.65	0.95	4.15	1.22	29.79	41.00	11.19
0.75	0.55	4.15	0.71	17.99	39.24	9.29
0.75	0.65	4.15	0.88	21.60	40.66	10.12
0.75	0.75	4.15	1.04	24.98	41.49	10.78
0.75	0.85	4.15	1.23	30.93	39.88	11.38
0.75	0.95	4.15	1.55	35.27	44.07	11.87
0.85	0.55	4.15	0.78	20.17	38.49	9.77
0.85	0.65	4.15	0.98	24.02	40.84	10.84
0.85	0.75	4.15	1.25	29.29	42.79	11.50
0.85	0.85	4.15	1.47	35.25	41.64	12.17
0.85	0.95	4.15	1.83	41.38	44.23	12.67
0.95	0.55	4.15	0.89	23.49	38.04	10.10
0.95	0.65	4.15	1.16	27.48	42.25	11.07
0.95	0.75	4.15	1.43	34.19	41.80	12.09
0.95	0.85	4.15	1.74	41.90	41.49	12.53
0.95	0.95	4.15	2.15	50.81	42.36	13.40
0.55	0.55	4.25	0.63	14.79	42.25	8.28
0.55	0.65	4.25	0.75	16.37	45.57	8.93
0.55	0.75	4.25	0.86	18.37	46.56	9.43
0.55	0.85	4.25	1.02	20.76	49.08	9.76
0.55	0.95	4.25	1.16	23.06	50.46	10.47
0.65	0.55	4.25	0.72	16.52	43.86	8.94
0.65	0.65	4.25	0.90	19.03	47.39	9.71
0.65	0.75	4.25	1.06	21.30	49.57	10.26
0.65	0.85	4.25	1.27	24.96	50.78	10.87
0.65	0.95	4.25	1.48	28.30	52.22	11.19
0.75	0.55	4.25	0.84	18.59	45.30	9.55
0.75	0.65	4.25	1.06	21.27	50.00	10.27
0.75	0.75	4.25	1.23	25.58	48.27	10.92
0.75	0.85	4.25	1.50	29.50	50.90	11.74
0.75	0.95	4.25	1.87	34.77	53.82	12.06
0.85	0.55	4.25	0.96	20.14	47.67	10.00
0.85	0.65	4.25	1.19	25.40	46.68	10.94
0.85	0.75	4.25	1.53	30.98	49.41	11.69
0.85	0.85	4.25	1.82	37.75	48.29	12.53
0.85	0.95	4.25	2.17	42.18	51.37	12.82
0.95	0.55	4.25	1.05	23.33	44.93	10.39
0.95	0.65	4.25	1.36	28.24	48.20	11.20
0.95	0.75	4.25	1.71	35.49	48.07	12.39
0.95	0.85	4.25	2.18	42.71	51.09	13.01
0.95	0.95	4.25	2.54	48.36	52.52	13.92

### The fundamental flipping frequency is a scaling factor of the system

**Figure 8:**
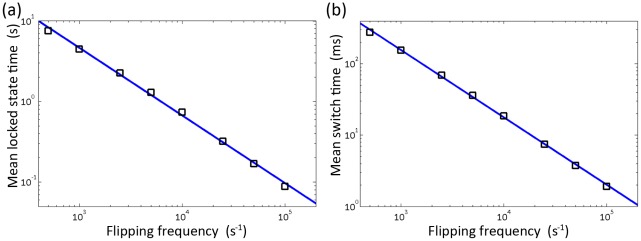
Dependence of A) mean locked state time and B) mean switch time on the fundamental flipping frequency *ω_a_*. The fundamental flipping frequency is a scaling factor of the system.

Although we have identified a best-fit parameter set that can well reproduce experimental findings, we have to note that these fit values are sensitive to the parameters fixed earlier, especially to the fundamental flipping frequency *ω_a_*. Here we investigate how mean locked state time and mean switch time respond to changes of *ω_a_* while other parameters remain fixed. We see in Figure 8 that the fundamental flipping frequency is a scaling factor of the system. Both mean locked state time and mean switch time are inversely scaled by the flipping frequency. When flipping frequency is higher, each protomer on the ring makes more attempts to flip to the opposite conformation and therefore the locked state becomes less stable (hence mean locked state time decreases) and transition becomes much faster (hence mean switch time decreases); when flipping frequency is lower, each protomer on the ring makes fewer attempts to flip to the opposite conformation and therefore locked state becomes more stable (hence mean locked state time increases) and transition becomes much shorter (hence mean switch time increases).

### Ring behavior at different sizes

In the above sections, we have determined the best-fit model parameters using experimental results. It will be interesting to test the ring behavior at different sizes using those values. When Duke et al. [15] first proposed the conformational spread model, they identified that *E_J_>k_B_T ln N* (N is the size of the ring) is the condition under which a large ring has the characteristic of a coherent switch. In the case of 34 protomers, this condition requires that *E_J_>3.5k_B_T*. When this condition is met, in time series of ring activity, we see for the majority of time that, the ring stays in complete active (active protomer = 34) or complete inactive (active protomer = 0) state. This invokes an empirical mathematical definition of ‘coherent switch’: the active number of protomers on the ring has to be in 0 or N for greater than 65% of the total simulation time.

**Figure 9:**
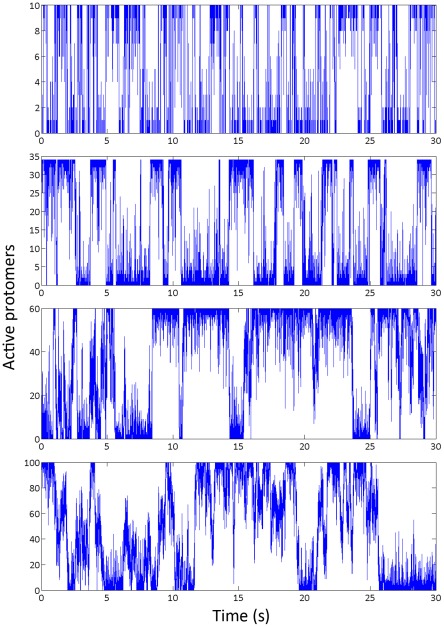
Ring behaviors at size of 10, 34, 60 and 100 protomers. The simulation time is set to 30 seconds, the number of active protomers shows locked state behavior and rapid switching.

We then simulated the ring activity at sizes of 10, 60, 100 protomers with activation energy *0.65 k_B_T* and coupling energy *4.15 k_B_T* at neutral bias and the result is shown in Figure 9. From the requirement of *E_J_>k_B_T ln N* we expect to see coherent switch behavior for ring sizes at 10, 34, and 60, but not at 100.

Indeed, in Figure 9, we see with the same parameter set, the smaller the ring is, the easier a switch happens. At ring size of 10, 34, 60 protomers, we see clear locked states in the trace and the switching events are fast. However, at the ring size of 100, a switch across the ring becomes very difficult and the time spent during a switch is comparable to the time the ring stays in a locked state. In Table 3, we made predictions about the mean locked state intervals, mean switch times and Hill coefficient of the switch response with activation energy *0.65 k_B_T* and coupling energy *4.15 k_B_T* at different ring sizes.

**Table 3:** Model predictions about motor switching time, locked state time, and Hill coefficient with activation energy *0.65 k_B_T* and coupling energy *4.15 k_B_T* at different ring sizes.

Size (protomers)	Mean Locked State time (s)	Mean Switch Time (ms)	Locked state / Switch time ratio	Hill coefficient
10	0.22	0.72	312.88	3.05
34	0.74	18.17	40.98	9.52
60	1.33	104.56	12.70	13.60
100	2.06	371.23	5.54	16.01

## Discussion

In this study, we undertook a comprehensive numerical simulation analysis of a general model of stochastic allostery in a protein ring and evaluated the ability of such a model to explain the switching, sensitivity and locked state behavior of the rotary bacterial motor. We modeled the gearbox of the motor as a ring of 34 identical protomers, a geometry inspired by the FliM structure in the motor complex, believed to be responsible for motor switching. The model is able to qualitatively reproduce the motor behavior, such as locked rotation in CCW or CW state and fast switching between the two. Furthermore, based on a comprehensive parameter space search, the model can also quantitatively account for the experimentally determined switch time, locked state interval and Hill coefficient of the motor. Specifically, we found a unique set of values that fit the experimental value best, activation energy must be around 0.65*k_B_T* and the cooperativity around 4.15*k_B_T*. The bounds around these values are tight. Smaller or larger energies result in rings that either (a) spend too long or too little time in the locked states, (b) do not have the required sensitivity or (c) far away from this parameter regime, fail to switch coherently.

With the ring operating in this parameter set, time traces of ring state (measured as the number of active protomers) indicate that the ring spends most of its time in one of the two locked states, with rare (every 0.5–2 seconds) switches between the two being accomplished very rapidly (on the order of milliseconds). The trace of ligand activity (measured as the number of ring protomers having bound ligand molecules) mirrors the ring state, with the two driving each other: binding of more ligand drives active domain formation, which in turn leads to a preference for having ligand bound and conversely. Rather than being completely locked in one stable state with all protomers being either active or inactive, the ring displays constant activity in the form of nascent domains of the opposite state to the locked state, seen as ‘noise’. For the vast majority of the time, only one such domain exists, and the presence of two (but no more) growing domains is frequently, but not always, associated with a switching event.

The model predicts that the time spent by the ring in the locked states corresponding to CW (all protomers active) and CCW (all protomers inactive) is exponentially distributed. The model can also predict the Hill coefficient (∼10) measured for the sigmoidal curve that relates CheY-P concentration to motor bias. Near the optimal parameter point identified, the distributions of the switching times are gamma-like with a peak around 5–8 ms.

To be effective, a switch must achieve two globally conflicting properties. It must accomplish sensitivity by amplifying small changes in the effector, but only over a narrow critical range (the switching point). Outside this range, it must accomplish reliability by being unresponsive to changes in the effector. The allosteric switching model explains how the motor simultaneously meets these competing design requirements. Near the critical CheY-P concentration, a highly cooperative mechanism (E_J_≫1*k_B_T*) is used to amplify small stochastically occurring nascent domains that can rapidly grow to encompass the entire switching complex. The resulting digital switch displays the desired selective ultrasensitivity but switches chaotically. In order to ensure switch reliability, CheY-P binding must moderately stabilize the protomer active state, providing a mechanism for biasing the whole switch complex by continuously varying the CheY concentration over a large range: a strategy typical of analog control. The values and ratio of the strengths of the two mechanisms must be tightly controlled in order for the switch complex to be functional. We hypothesize that this control is accomplished through the biochemical structure of the protomers and ring, which are genetically determined and so robust to intracellular noise during the cell's life. In light of recent studies of digital cellular signaling [20], we wish to further suggest that the combination of analog and digital control here proposed to explain the behavior of the bacterial switch complex may be a motif typical of biological switch design.

In this study we have focused on a ring consisting of 34 protomers because it is believed that the C-ring in the *E. coli* flagellar motor, which consists of 34 copies of the protein FliM, acts as the motor direction switch. However, numerous examples of protein rings and other interconnected protein complex geometries are known, including DNA polymerase sliding clamps, voltage-gated ion channels, ATP synthase etc. Each of these rings may hypothetically accomplish its function using a conformational spread mechanism, but would consist of different numbers of protomers. In our model this can be simulated by changing *N*, the number of elements in the ring. In this paper, we have narrowed our study to a closed protein ring. However, we have to point out that the conformational spread model as well as the numerical method we presented here can be easily modified to describe one dimensional allostery regulation in a protein chain or a strand of DNA molecules. The model can also be modified to describe signal transduction and amplification on a two dimensional plane, which will be of great use in studying functions of cellular receptors.

Allostery is a widespread mechanism in biology and conformational change is the basis for a large subset of all protein function. Since protein complexes are the workhorses of the cell, we expect models similar to this and the idea of conformational spread in general to be increasingly important in systems biology and biophysics. Investigating the applicability of conformational spread models to other biological systems will be the subject of future work.

Bacterial chemotactic exploration depends on the ability of the flagellar motors at the base of the flagella to perform two tasks: (1) remain stable in their current direction of rotation for long periods (seconds) as required and (2) switch quickly between the two directions in response to the environmental changes detected by the chemotaxis pathway. These properties make the bacterial switch an exquisite computational element that combines ultrasensitivity and reliability. In this paper we presented an analysis of a model featuring conformational spread that aims to explain the mechanism of the motor switch. Simulations confirm that this model is able to reproduce the characteristics of the motor observed in experiments. We speculated that stochastic models of conformational spread will be a common theme in protein allostery and signal transduction.
